# Innate and Adaptive Immune Parameters following mRNA Vaccination in Mice

**DOI:** 10.3390/vaccines12050543

**Published:** 2024-05-15

**Authors:** Srinivasa Reddy Bonam, Nicholas C. Hazell, Mano Joseph Mathew, Yuejin Liang, Xuxiang Zhang, Zhi Wei, Mohamad-Gabriel Alameh, Drew Weissman, Haitao Hu

**Affiliations:** 1 Department of Microbiology and Immunology, University of Texas Medical Branch, Galveston, TX 77555, USA; nchazell@utmb.edu (N.C.H.); yu2liang@utmb.edu (Y.L.); 2 Department of Pathology, University of Texas Medical Branch, Galveston, TX 77555, USA; 3 EFREI Research Lab, Panthéon Assas University, 30-32 Avenue de la République, 94800 Villejuif, France; mano.mathew@efrei.fr; 4 Laboratoire Génomique, Bioinformatique et Chimie Moléculaire, EA7528, Conservatoire National des Arts et Métiers, HESAM Université, 2 rue Conté, 75003 Paris, France; 5 Department of Computer Science, New Jersey Institute of Technology, Newark, NJ 07102, USA; xz445@njit.edu (X.Z.); zhiwei04@gmail.com (Z.W.); 6 Perelman School of Medicine, University of Pennsylvania, 3400 Civic Center Blvd, Philadelphia, PA 19104, USA; mg.alameh@pennmedicine.upenn.edu (M.-G.A.); dreww@pennmedicine.upenn.edu (D.W.); 7 Institute for Human Infections and Immunity, University of Texas Medical Branch, Galveston, TX 77555, USA; 8 Sealy Institute for Vaccine Sciences, University of Texas Medical Branch, Galveston, TX 77555, USA

**Keywords:** innate immunity, adaptive immunity, mRNA vaccine, lipid nanoparticles

## Abstract

The COVID-19 pandemic has raised the standard regarding the current vaccine development pace, as several messenger RNA (mRNA)-lipid nanoparticle (LNP) vaccines have proved their ability to induce strong immunogenicity and protective efficacy. We developed 1-methylpseudouridine-containing mRNA-LNP vaccines, expressing either the more conserved SARS-CoV-2 nucleoprotein (mRNA-N) or spike protein (mRNA-S), both based on the prototypic viral sequences. When combining both mRNA-S and mRNA-N together (mRNA-S+N), the vaccine showed high immunogenicity and broad protection against different SARS-CoV-2 variants, including wildtype, Delta, BA.1, BA.5, and BQ.1. To better understand the mechanisms behind this broad protection obtained by mRNA-S+N, we analyzed innate and adaptive immune parameters following vaccination in mice. Compared to either mRNA-S or mRNA-N alone, mice vaccinated with mRNA-S+N exhibited an increase in the innate immune response, as depicted by the higher cytokine (IL-6 and chemokine (MCP-1) levels. In addition, lymph node immunophenotyping showed the maturation and activation of dendritic cells and natural killer cells, respectively. To understand the adaptive immune response, RNA-Seq analyses of the lung and spleen samples of the vaccinated mice were performed in parallel and revealed a stronger immune gene-expression profile in the lung than that in the spleen. Compared to mRNA-S alone, mRNA-S+N vaccination elicited higher levels of expression for genes involved in multiple immune pathways, including T cells, cytokine signaling, antigen presentation, B cells, and innate immunity. Together, our studies provide immunological insights into the mechanisms of broad protection conferred by dual mRNA vaccination against SARS-CoV-2 variants.

## 1. Introduction

The emergence of COVID-19 expedited the use of messenger RNA (mRNA) lipid nanoparticle (LNP) technology in vaccine development. The two efficacious COVID-19 mRNA-LNP vaccines, BNT162b2 (Pfizer-BioNTech) and mRNA-1273 (Moderna), had a significant impact on the development of several additional platforms treating infectious diseases, autoimmune diseases, cancer, and others [1,2]. As several mRNA-LNP platforms (across different groups) are being developed for mRNA delivery, each composition is unique; thus, a better understanding of their functionality is needed for validation [1,2]. Although mRNA-based vaccines were introduced in the early 1970s, several challenges, including the delivery of an mRNA drug substance to its pharmacological site of action (i.e., cytosol) provided limitations until the development of the potent lipid-based delivery system (LNP) [1,2,3]. In addition, mRNA is intrinsically sensed by the endogenous pattern recognition receptors (PRRs), another important limitation. Several discoveries have played a major role in improving exogenous mRNA’s ability to evade endogenous PRRs after mRNA immunization. By replacing standard uridine nucleotides with chemically modified uridines, adding a Cap1 structure to the 5′ end and removing double-stranded RNA contaminants from the in vitro-transcribed (IVT) mRNA rendered naked mRNA non-inflammatory and physically stable [1,4]. 

In addition, LNPs can further increase the chemical and/or biological stability of mRNA, resulting in an efficient mRNA delivery system. Indeed, IVT-mRNA carried by LNPs is a possible candidate for activating the immune system. We generated a nucleoside-modified mRNA-LNP vaccine that encodes full-length Severe Acute Respiratory Syndrome Coronavirus 2 (SARS-CoV-2; Wuhan-Hu-1 strain) nucleoprotein (N) (mRNA-N) or spike (S) (hereafter mRNA-S). Our previous studies, using mRNA-N or mRNA-S either alone or in combination (mRNA-S+N), confirmed the immunogenicity and efficacy of mRNA vaccines against SARS-CoV-2, including wildtype and variants of concern (VOCs) [5]. A growing number of studies are available on the innate immune response induced by mRNA-LNP vaccines, with varying results [6]. A clear picture of the innate and adaptive immune mechanisms elicited by our mRNA vaccines, which lead to broad protection against SARS-CoV-2 variants, has yet to be elucidated. After the delivery of mRNA into the cell by LNPs, mRNA encounters several important cellular PRRs, such as TLR-3, TLR-7, and TLR-8 [5,7,8]. Recent studies indicate that LNPs have the ability to stimulate the immune system [9,10,11]. The adjuvant activity of the LNPs is dependent on its composition, particularly the ionizable lipid structure [4,7,9,12]. Emerging evidence suggests that ionizable lipids themselves are recognized as non-self-molecules by PRRs [12,13]. 

On the other hand, mRNA-LNP-induced adaptive immunity is a vital factor in determining the vaccines’ efficacy. In addition to antibody responses (both binding and neutralizing), COVID-19 mRNA vaccines induce antigen-specific CD4^+^ T cells, CD8^+^ T cells (including IFN-γ-secreting CD8^+^ T cells) and T follicular cells, which are detectable up to 6 months post-boost-vaccination in humans [3,14,15]. Initial mouse studies with our mRNA vaccines have shown that mRNA-N is highly immunogenic and induces strong N-specific binding antibody and T cell (both CD4^+^ and CD8^+^) responses, despite there being no neutralizing antibodies. Although only modest effects were achieved, mRNA-N vaccination did protect the mouse and hamster from mouse-adapted SARS-CoV-2 and VOCs, respectively [5,16]. Critically, our study also showed that combined mRNA-N and mRNA-S immunization (mRNA-S+N) protected against Delta and Omicron infection in hamsters, whereas mRNA-S alone only induced mild protection [5]. The CD8^+^ T cell depletion experiment supports the idea that cellular immunity plays a critical role in mRNA-S+N’s protection against SARS-CoV-2 variants [5]. Despite these earlier findings, the innate and adaptive immune profiles elicited by dual mRNA-S+N as compared to the use of mRNA-S alone remain to be further elucidated. 

In this brief study, we aim to understand both the early- and late-phase events stimulated in our mRNA vaccines. As such, we determined the innate and adaptive immunity gene signatures induced by LNP, mRNA-S, mRNA-N, or mRNA-S+N post vaccination. All vaccine-treated mice, including empty LNP (eLNP), can effectively induce the innate immune response evidenced by detectable serum IL-6 and MCP-1. A retrospective analysis revealed that the chemokine/cytokine production of LNPs showed a good correlation with their antibody production. In addition to humoral and cell-mediated immune response data, a parallel RNA-seq analysis of the lung and spleen samples of the immunized mice revealed that a stronger immune gene-expression profile in the lung was observed compared to the spleen, which further supports our earlier respiratory protection study against SARS-CoV-2 VOCs. 

## 2. Materials and Methods

### 2.1. Study Design

The aim of the present study is to evaluate the innate immune response of either LNPs, and/or LNPs encapsulating mRNA-S or mRNA-N or mRNA-S+N in mice. We previously confirmed mRNA-N and mRNA-S protein expression by 293T cells, and the results are published in our previous study [5]. The animal study protocols at the University of Texas Medical Branch (UTMB), with protocol numbers 1703020A and 2009087, were approved by the Institutional Animal Care and Use Committee. We performed animal research in accordance with the standards outlined in the National Institutes of Health’s Guide for the Care and Use of Laboratory Animals. The sample size (n = 6) for each animal investigation was set without employing a power analysis. Instead, it was established using data from earlier studies conducted in our laboratory and those documented in the previous literature.

### 2.2. mRNA Synthesis, LNP Formulation, and Confirmation of Protein Expression

The mRNA synthesis was carried out and formulations of LNP were created in accordance with prior protocols [5]. Briefly, mRNA-N (full-length) and S (prefusion-stabilized S protein with two proline mutations; mRNA-S-2P) encoding our target proteins were synthesized using the T7 RNA polymerase (MegaScript, Ambion, Austin, TX, USA) IVT kit. The sequences are based on the ancestral SARS-CoV-2 Wuhan-Hu-1 strain (GenBank MN908947.3). To synthesize nucleoside-modified mRNAs, one-methylpseudouridine (m1)-5′-triphosphate was substituted for uridine triphosphate. Additionally, to enhance protein expression, polyadenylated tails were appended to the termini of these modified mRNAs. The ScriptCap m7G capping system and ScriptCap 2′-O-methyltransferase kit were employed to encapsulate IVT mRNAs, followed by cellulose-based purification. The mRNAs were formulated into LNPs using an ethanolic lipid mixture (ALC-0315:DSPC:Cholesterol:DMG-PEG2000 = 50:10:38.5:1.5) and an mRNA containing an aqueous buffer system. mRNA-LNPs were prepared in accordance with RNA concentrations (1 µg/µL) and stored at −80 °C until use. Both N and S protein expression were confirmed by transfecting mRNAs into 293T cells. After 24 h, mRNA-N and S protein expression were determined by Western blot and flow cytometry, respectively. The detailed methodology is described in our recent publication [5].

### 2.3. Immunization

After acclimating, mice were divided into two experimental setups: innate immune groups and adaptive immune groups. For innate immune experiments, female Balb/c mice were immunized with either PBS or LNP (2 µg), mRNA-S (2 µg), mRNA-N (2 µg), or mRNA-S+N (1 µg each) intramuscularly (IM) on the right thigh region. The same dose was used for each group to ensure comparable amounts of mRNA or LNP were administered. Blood was collected via the retro-orbital route and serum was harvested 8 h post immunization. Subsequently, terminal harvest was conducted at 24 h after immunization to collect serum for measuring cytokines/chemokines and lymph nodes were collected for immune cell phenotyping. For adaptive immune response experiments, Balb/c mice were immunized at day 0 with either LNP (1 µg), mRNA-S (1 µg), mRNA-N (1 µg), or mRNA-S+N (1 µg each), followed by a booster at day 21 on the left thigh region. At two weeks post booster, mice were sacrificed and both spleen and lung were harvested for RNA-Seq.

### 2.4. Cytokine/Chemokine Assay

Blood samples collected from the immunized mice were incubated at room temperature for 1 h, followed by centrifugation at 5000 rpm for 10 min at 4 °C. Twenty-five microliters of serum were subjected to bead-based cytokine and chemokine assay (LEGENDplex mouse inflammation panel, BioLegend, San Diego, CA, USA) according to the manufacturer’s recommendations. The analysis was performed on a BD FACSymphony machine (BD Biosciences, Milpitas, CA, USA) using the LEGENDplex data analysis software (BioLegend online software).

### 2.5. In Vivo 

To determine the LNPs’ tissue distribution, firefly luciferase mRNA (FLuc mRNA) was encapsulated by LNPs. FLuc mRNA-LNPs were injected in 6–8-week-old C57BL/6J mice (n = 1 untreated and 2 FLuc mRNA-LNPs treated) via the IM route at a dose of 0.25 mg/kg body weight. At 6 h post administration, 1.5 mg/mice of D-luciferin substrate was administered by the intraperitoneal route (250 µL). At 15 min post substrate administration, mice were sacrificed, with the heart, lung, liver, spleen, and kidneys collected. Luciferase expression was acquired by using an IVIS Spectrum (Perkin Elmer, Waltham, MA, USA).

### 2.6. Lymph Node Immunophenotyping

Mice inguinal draining lymph nodes were harvested 24 h after immunization, homogenized with a syringe plunger, and filtered through a 70 mm cell strainer on ice. After a PBS wash, the cells were washed with FACS buffer (1% FBS in 1× PBS). Furthermore, they were subjected to counting and surface staining in Fc buffer (10% FBS in 1× PBS) using the following antibodies for 30 min on ice. CD3e-BUV395 (Clone: 145-2C11), CD8a-PerCp/Cy5.5 (Clone: 53–6.7), CD4-FITC (Clone: GK1.5), CD11b-BV421 (Clone: M1/70), CD11c-BV786 (Clone: HL3), CD80-BV650 (Clone: 16-10A1), CD206-BV605 (Clone: C068C2), I-A/I-E-AF700 (Clone: M5/114.15.2), CD49b-APC (Clone: DX5), CD86-PE/Cy7 (Clone: GL-1), CD83-BUV805 (Clone: Michel-19), Ly-6G-BV711 (Clone: 1A8), CD335 (NKp46)-PE (Clone: 29A1.4). Fixed viable dye-e506 was used to discriminate between live and dead cells. After the incubation, cells were processed for surface staining for various markers, acquired using an BD FACSymphony A5 SE (BD Biosciences, Franklin Lakes, NJ, USA), and the data were analyzed by FlowJo software (v11).

### 2.7. RNA Extraction, Library Construction, High-Throughput Sequencing, and Transcriptome Analysis

Spleen and lungs were maintained in DMEM containing 1% pen-strep and 2% fetal bovine serum before tissue homogenization using sterile, 5 mm, stainless steel beads. The supernatants from the homogenates were dissolved in the trizol-LS reagent, with RNA extracted according to the manufacturer’s instructions. RNA extracted from lung and spleens were subjected to bulk RNA sequencing analysis. The libraries for deep sequencing were created using the Smart-3SEQ procedure, as described in a previous publication [17]. The Smart-3SEQ protocol adds a five-base unique molecular identifier (UMI) and 3 Gs to the 5′ end of each sequence. These were removed from the reads and the UMI was added to the read name with the umi_homopolymer.py software provided by the Smart-3SEQ authors (https://github.com/jwfoley/3SEQtools) (accessed on 7 March 2023). Reads were aligned to the Mus musculus NCBI assembly GCF_000001635.27 (GRCm39) using STAR version 2.7.9a with the parameters recommended by the software authors for the Encode consortium. We used Nextflow: 22.10.7, nf-core/rnaseq: 3.10.1 and Salmon 1.9.0 for quantification. The quantification table was utilized as an input in DESeq2 for estimating differential gene expression, following the guidelines provided in the DESeq2 vignette accompanying the software. The genes were hierarchically clustered using the heatmap program in R. We acknowledge our use of the gene set enrichment analysis, GSEA software (v4.3.3), and Molecular Signature Database (MSigDB) [18] (https://www.gsea-msigdb.org/gsea/index.jsp) (accessed on 5 May 2023), following the vignettes provided by the authors.

### 2.8. Statistical Analysis

Statistical analyses were performed using GraphPad Prism version 9.4.1, and the type of statistic applied and the level of statistical significance are described in the respective figure legends. Non-parametric tests were employed for statistical analysis throughout this study. The data are shown as the mean with a 95% confidence interval. A comparison was performed among groups using one-way ANOVA, followed by a Kruskal–Wallis test and Dunn’s multiple comparisons test. *p* values are denoted with *p*, and values <0.05 were considered significant.

## 3. Results

### 3.1. mRNA-LNP Vaccination in Mice Induced Innate Immune Response

**Figure 1:**
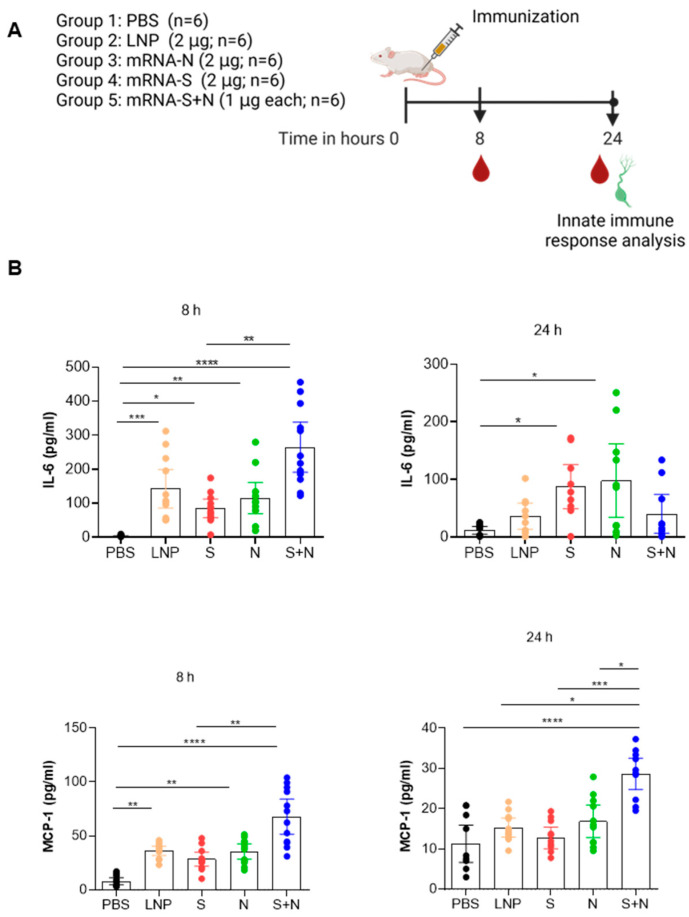
mRNA-LNP vaccination in mice induces a robust innate immune response. (**A**). Balb/c mice were injected with either LNP (2 µg), mRNA-S (2 µg), mRNA-N (2 µg), or mRNA-S+N (1 µg of each) intramuscularly. (**B**). Serum cytokines were measured after 8 and 24 h; n = 4–12. Data are presented as mean with 95% confidence interval. Nonparametric test, Kruskal–Wallis, and One-Way ANOVA, along with Dunn’s multiple comparisons post hoc test, were used for statistical analysis. * *p* < 0.05, ** *p* < 0.01, *** *p* < 0.001, and **** *p* < 0.0001.

We previously evaluated our mRNA-LNP vaccine’s immunogenicity and efficacy in Balb/c and Syrian hamster models [5]. However, the innate immune response of our vaccines was not elucidated. In this study, we further evaluated the innate immune response of our vaccines in the Balb/c mouse model. Mice were immunized IM with either PBS (mock control), empty LNP (eLNP), mRNA-S, mRNA-N, or mRNA-S+N, with the dose based on our previous data [5]. At 8 and 24 h post injections, we assessed the levels of serum cytokines and chemokines using the LEGENDplex mouse inflammation panel, which consists of interleukin (IL)-1α, IL-1β, IL-6, IL-10, IL-12p70, IL-17α, IL-23, IL-27, granulocyte-macrophage colony stimulating factor (GM-CSF), interferon (IFN)-β, IFN-γ, monocyte chemoattractant protein (MCP)-1, and tumor necrosis factor-α (TNF-α) (Figure 1). We found that the vaccines, including eLNP, significantly induced the secretion of IL-6 and MCP-1 at both 8 and 24 h compared to PBS group. In addition, mRNA-S+N revealed a more profound increase in the IL-6 and MCP-1 production as compared to mRNA-S or mRNA-N alone. Interestingly, at 8 h post injection, the mRNA-S vaccine alone showed a transient increase in IL-1α production, which became less evident 24 h after immunization. Unlike IL-6 and MCP-1, there was little or no increase in the levels of other major inflammatory cytokines, such as IL-1β, TNF-α, IFN-β, IFN-, and IL-27 (Figure S1). In addition, a comprehensive analysis of the phenotypes of immune cells in draining lymph nodes (dLNs) was conducted (see the gating strategy; Figure S2A,B). The analysis of immune cells in the dLNs of vaccinated mice showed an increase in dendritic cells (DCs) and natural killer (NK) cell populations (Figure S2C). Compared to other treatment groups, mRNA-S+N showed significantly higher levels of matured DCs, indicated by the expression of CD86 (Figure S2C), in correlation with other laboratory reports [9]. Other innate immune cells were recruited to the dLNs (based on their number); however, there is no significant difference (Figure S2A,B).

### 3.2. mRNA-N Vaccination Regulates both Innate and Adaptive Immune Transcripts

**Figure 2:**
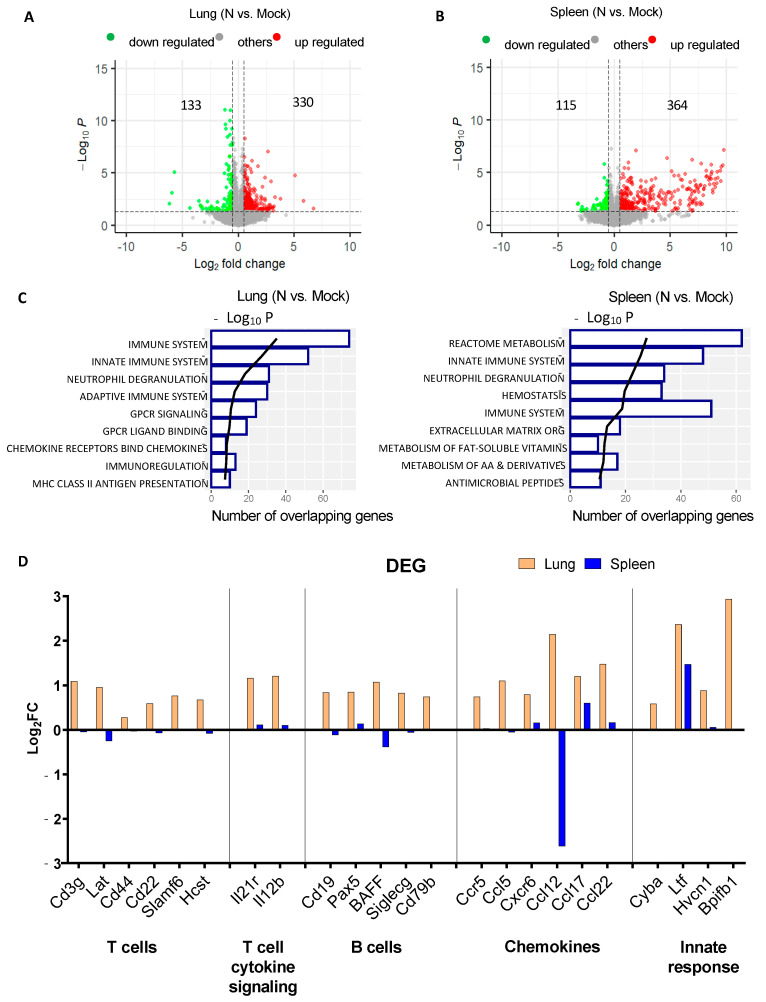
Mouse lung and spleen RNA-Seq analysis after mRNA-N vaccination. (**A**,**B**) Volcano plots showing global DEGs in mouse lungs (**A**) and spleens (**B**) following mRNA-N vaccination compared to mock group (n = 4 per group). Red and green dots indicate upregulated and downregulated genes, respectively. The *x*-axis shows the magnitude of the change as log_2_-fold change in the gene expression, and the *y*-axis shows the statistical significance of the change as log_10_ *p*-value. (**C**) Gene set enrichment analysis of DEGs in lungs and spleens. Horizontal bars indicate the number of DEGs identified for each pathway, as delineated by the lower *x*-axis. Black trend lines represent ranking by *p* value (−log_10_ [p-adjust]) in the upper *x*-axis. (**D**) Log_2_-fold change in selected DEGs in the lungs (orange) or in the spleens (blue) of mice vaccinated with mRNA-N relative to the mock control.

Compared to the hamster model, the mouse model provides advantages for immunological and mechanistic investigations of vaccines due to the vast availability of the research tools. As mentioned above, we previously examined the immunogenicity of different mRNA vaccines in Balb/c mice, including mRNA-N alone, as a T-cell based vaccine that provides partial but significant protection against SARS-CoV-2 variants [5]. To gain a more in-depth mechanistic insight into host respiratory and systemic immune responses following mRNA vaccination, Balb/c mice were respectively vaccinated via the IM route with mRNA-N (1 µg/dose) or eLNP at weeks 0 and 3, followed by collection of the lung and spleen at week 5 for bulk RNA-Seq analysis for assessing airway and systemic transcriptomic profiles (Figure S3A). Compared to the mock control, mRNA-N vaccination induced notable changes in transcriptomic profiles in the lung, as well as in the spleen. In the lung of mRNA-N-immunized mice, a total of 330 genes (out of 16315) were significantly upregulated, with 133 genes significantly downregulated, compared to our mock group (Figure 2A). Comparable numbers of differentially expressed genes (DEGs) were observed in the spleen: 364 genes were upregulated, and 115 genes were downregulated in the vaccinated mice relative to the mock group (Figure 2B). Gene enrichment analysis showed that, in the lung, the top pathways upregulated by mRNA-N vaccination are involved in the immune response, innate immune system, neutrophil degranulation, adaptative immune system, and signaling by G-protein-coupled receptors. In contrast, the top pathways upregulated by the vaccine in the spleen included a more diverse set of functions, including the metabolism, innate immune system, neutrophil degranulation, and hemostasis, indicating the dominant activation of immune pathways by the vaccine in the lung as compared to the spleen (2 weeks after booster) (Figure 2C). To gain more insight into the DEGs, we focused our analysis on individual genes and found that those involved in T-cell response (CD3g, Lat, CD44, CD22, Slamf6, and Hcst), T-cell cytokine signaling (IL21r and IL12b), B cell response (CD19, Pax5, BAFF, Siglecg, CD29b), and chemokine signaling (CCR5, CCL5, CXCR6, CCL17, and CCL22) were significantly upregulated in the lung, but not the spleen (Figure 2D). These data indicate that mRNA-N vaccination elicits a gene signature of immune response in the lungs.

### 3.3. mRNA-S+N Vaccination Induces Stronger Innate and Adaptive Immune Profiles than mRNA-S

**Figure 3:**
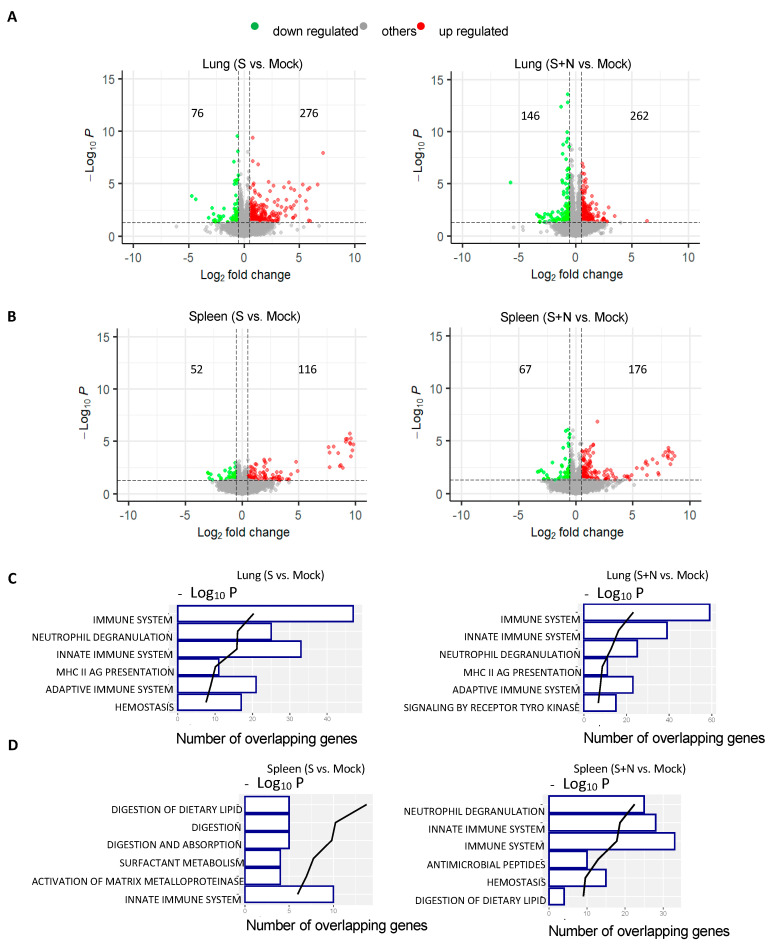

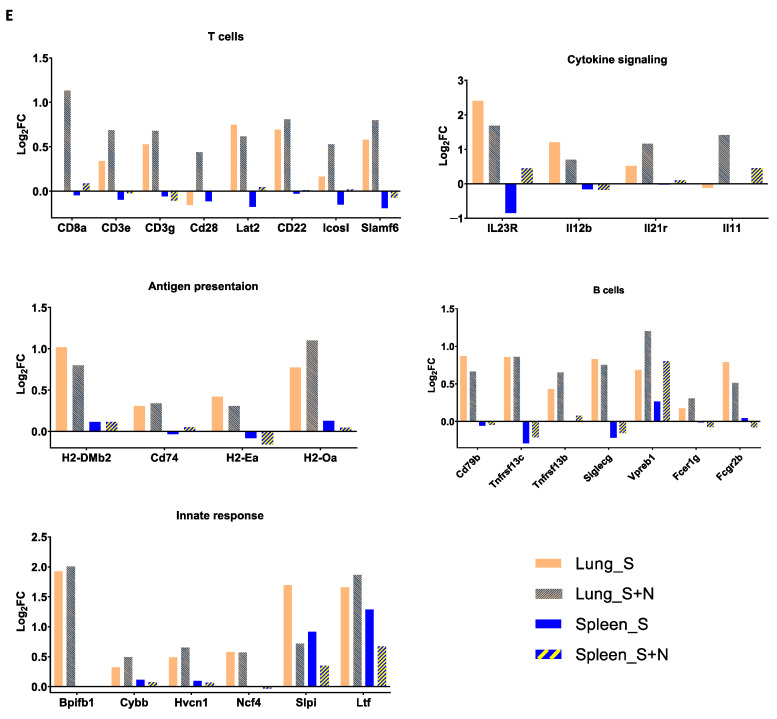
Transcriptomic analysis of mouse lungs and spleens following vaccination with mRNA-S alone or dual mRNA-S+N. (**A**,**B**) Volcano plot representation of DEGs induced by mRNA-S alone and by dual mRNA-S+N in the lungs (**A**) and spleens (**B**). Red dots represent the significantly upregulated genes, and green dots indicate the significantly downregulated genes. The *x*-axis shows log_2_ fold change in gene expression and *y*-axis shows log_10_ *p*-value for each gene. (**C**,**D**) Gene set enrichment analysis of DEGs in lungs (**C**) and spleens (**D**). Numbers in the *x*-axis show the number of genes that overlapped in each pathway. Black trend line represents ranking by *p* value (−log_10_). (**E**) Relative expression of listed DEGs regulated by mRNA-S alone or by mRNA-S+N (Log_2_ FC to the mock control) in the lungs (1st and 2nd yellow bars) and in the spleens (3rd and 4th blue bars).

To characterize global respiratory and systemic transcriptomic changes following dual mRNA-S+N vaccination as compared to mRNA-S alone, Balb/c mice (n = 4) were vaccinated with eLNP (mock), mRNA-S, or mRNA-S+N at weeks 0 and 3, followed by the collection of lung and spleen tissues at week 5 for RNA-Seq analysis (Figure S3B). The mock group (n = 4) was used for normalization. In the lung, mRNA-S vaccination led to 276 genes being upregulated and 76 genes being downregulated relative to the mock control, and dual mRNA-S+N vaccination induced the regulation of a higher number of DEGs, including 262 genes that were upregulated and 146 genes that were downregulated (Figure 3A). Compared to the lung, both immunizations induced lower numbers of DEGs in the spleens (Figure 3B). A gene enrichment analysis of DEGs showed that the top pathways induced by mRNA-S alone and by dual mRNA-S+N in the lung were similar and related to host immunity, including the immune system, innate immune system, neutrophil degranuation, MHC-II antigen presentation, and adaptive immune system (Figure 3C), indicating the induction of a strong immune gene signature in the lung by mRNA vaccination. The mRNA-S alone and mRNA-S+N differed in the sixth-ranked pathway they regulated: hemostasis by mRNA-S and tyrosine kinase receptor signaling by mRNA-S+N (Figure 3C). While both mRNA-S alone and mRNA-S+N elicited similar immune pathways in the lungs, the transcriptomic changes associated with these pathways by mRNA-S+N appeared to be more profound than those exhibited by mRNA-S alone, based on the number of regulated genes (Figure 3C; *x*-axis) and the *p* value (Figure 3C; *y*-axis). By contrast, at 2 weeks post booster vaccination, minimal immune gene signatures were detected in the spleen by mRNA-S alone (Figure 3D), whereas mRNA-S+N vaccination led to a significant expression of immune genes in the spleen (Figure 3D), with the top three pathways identified as neutrophil degranulation, immune system, and the innate immune system (Figure 3D). Overall, vaccination with mRNA-S and mRNA-S+N elicited a stronger signal of immune gene expression in the lung than in the spleen at 2 weeks post booster (Figure 3A–D). We further evaluated individual DEGs and found that the genes involved in T-cell response (CD8a, CD3e, CD3g, Lat2, CD22, lcosl, and Slamf6), cytokine signaling (IL23R, IL12b, IL21r, and IL11), antigen presentation (H2-DMb2, CD74, H2-Ea, and H2-Oa), B cell response (CD79b, Tnfrsf13c, Tnfrsf13b, Siglecg, Vpreb1, Fcer1g, and Fcgr2b), and innate immune response (Bpifb1, Cybb, Hvcn1, Ncf4, Slpi, and Ltf) were significantly upregulated in the lung by both mRNA-S and mRNA-S+N as compared to the mock control (Figure 3E). Some genes, especially CD8a, CD3e, CD28, and ICOSl, were upregulated by mRNA-S+N at higher levels than those by mRNA-S (Figure 3E). In contrast, significant upregulation of these immune genes was not observed in the spleen of the same animals (Figure 3E). Together, the data further support that dual mRNA-S+N elicits an immune-related gene expression profile in the lung at 2 weeks post booster immunization, which is less evident in the spleen.

## 4. Discussion

The mRNA SARS-CoV-2 vaccines were brought to market in response to the COVID-19 public health crisis. The continuous evolution and spread of VOCs still pose a major threat to the public health around the world and a pan-COVID-19 vaccine which can maintain efficacy across VOCs is still needed. Therefore, developing an efficacious mRNA-LNP vaccine and understanding its immunogenicity potential is still of prime importance. 

We developed mRNA-LNP vaccines expressing either S or N targets derived from the prototypical virus. Our combination mRNA-S+N strategy successfully protected hamsters against all SARS-CoV-2 VOCs (Delta and BA.1 published elsewhere; BA.5 and BQ.1 under communication) we have examined so far. Nevertheless, their innate and adaptive immune mechanisms are only partly explored. Although several research groups have elucidated the innate immune responses of mRNA-LNPs, each LNP’s composition is unique [2,9,13,19,20,21,22]. Interestingly, eLNPs are globally regarded as vaccine adjuvants [23,24,25,26]. For example, eLNPs co-injected with the H1N1 antigen induced robust antibody production against H1N1, comparable to Alum-based adjuvants [27]. The antibody production is mediated by the strong T follicular helper cell, germinal center B cell, long-lived plasma cell, and memory B cell responses [28]. Recent studies on mice revealed that the injection of eLNPs (either topical, IM, or intranasal) induces the secretion of various cytokines and chemokines, which help in the infiltration of neutrophils and monocytes at the injection site [4,29]. In addition, the similar mechanism of the innate immune response was confirmed on human cells. The effect of eLNP treatment on monocyte-derived dendritic cells resulted in a maturation of signaling via the upregulation of CD40 followed by cytokine secretion. Interestingly, the phosphorylation of TANK binding kinase 1 (pTBK1) and interferon response factor 7 (pIRF7) is the downstream signaling of monocyte-derived DC maturation [9,30]. In agreement with the above results, we also witnessed an increase in mature DCs and activated NK cells in the lymph nodes. Moreover, an earlier study revealed that the adjuvant activity of eLNPs almost completely disappears when ionizable lipids are removed from their lipid composition. This observation was a large part of the basis for the hypothesis that ionizable lipids are critical for the adjuvant activity of LNPs. The number of reports on the adjuvant activity of LNPs in RNA vaccines is growing [9,13]. As a result, the recruited innate immune cells capture the LNPs and process them before their internalization. An optimal LNP composition is also known to increase the RNA uptake by splenic cells and to enhance the activation of innate immune responses by significantly inducing IL-6, CXCL1, CCL2, and CXCL10 [8]. Chemokines and cytokines are involved in the recruitment of monocytes and neutrophils. These observations support that LNPs do indeed function as an adjuvant in RNA vaccines. We also witnessed the localization of LNPs in the spleen 6 h post IM administration (Figure S4), indicating that our LNPs behave in a similar manner. Notably, our study showed that IL-6 and MIP-1 are the two major cytokine/chemokines induced by our mRNA vaccines, which is consistent with prior reports [28]. In our model, other major cytokines, including TNF-a, IL-1b, and type-I IFN, were not detected at all. This is likely because our mRNA-LNPs do not stimulate the production of these cytokines or are at low levels that are undetectable by the biplex assay. Nevertheless, a better understanding of the innate immune mechanisms that govern induction of adaptive immunity and vaccine efficacy in mRNA-LNP vaccination is an important topic and warrants further future investigation.

A previous transcriptomic analysis of vaccination, including mRNA vaccination, focused on the innate immune response [31,32]. Our study analyzed the transcriptional profiles of both lungs and spleens in parallel, using bulk RNA-Seq, from the same vaccinated animals (n = 4) 2 weeks after booster immunization, with the goal of understanding mRNA vaccine-induced respiratory and systemic immune gene signatures globally. Our data led to several observations. First, all mRNA vaccinations (mRNA-S, N, and S+N) led to a gene expression profile predominantly related to host immunity in the lung, which is less evident in the spleen at 2 weeks post booster immunization. These data indicate the induction of airway immune response and are consistent with prior reports on the mRNA vaccine-induced expansion of T cells in multiple tissues, including mouse and human airway tissues [33]. Second, the transcriptomic signature includes the upregulation of a range of DEGs involved in T-cell function, cytokine signaling, and antigen presentation, which is consistent with our earlier report showing the induction of strong CD4^+^ and CD8^+^ T-cell responses by both mRNA-N and mRNA-S [5]. Compared to mRNA-S alone, mRNA-S+N immunization elicits stronger immune gene signatures in the lungs and higher levels of expression for CD8a and CD3e genes (Figure 3C–E). The RNA-seq data indicate the induction of a strong respiratory CD8 T-cell response by mRNA-S+N, which is consistent with our previous report [5] and may correlate with its broad protection against SARS-CoV-2 variants. Indeed, our previous study confirmed the role of CD8 T cells in protection against SARS-CoV-2 Delta and Omicron BA.1 using in vivo cell depletion [5]. Lastly, in addition to the adaptive immune signatures, our data show a gene signature related to neutrophil activation following mRNA vaccination, although no significant difference was detected between the mRNA-S and mRNA-S+N groups. The role of neutrophil activation in protection against SARS-CoV-2 following mRNA vaccination is not yet clear and warrants investigation in future studies. 

## 5. Conclusions

Serum and immunophenotyping analyses of lymph node demonstrated the induction of innate immunity by our mRNA-LNP vaccines, especially the induction of IL-6 and MCP-1. RNA-Seq analysis revealed the induction of strong transcriptomic signatures by the mRNA-S+N vaccine in the lungs, leading to high levels of expression for genes involved in multiple adaptive immunity pathways. Together, our studies provide immunological insights into the mechanisms of broad protection conferred by mRNA-S+N.

## Data Availability

Data can be obtained upon request from the corresponding authors.

## Supplementary Materials

The following supporting information can be downloaded at: https://www.mdpi.com/article/10.3390/vaccines12050543/s1, Figure S1: mRNA-LNP vaccination in mice induces robust innate immune response; Figure S2: Immune phenotyping of lymph nodes from mRNA-LNP vaccinated mice; Figure S3: mRNA-LNP vaccination design; Figure S4: In vivo FLuc mRNA-LNP delivery.
