# Supplementary material for: Innate and Adaptive Immune Parameters following mRNA Vaccination in Mice
Source: Vaccines (Basel). 2024 May 15;12(5):543. doi: 10.3390/vaccines12050543 (PMC11125668; doi:10.3390/vaccines12050543)
Supplement: Supplementary file 1 [file vaccines-12-00543-s001.zip › vaccines-2986842-supplementary.pdf]

Communication

Innate and adaptive immune parameters following mRNA vaccination in mice

Srinivasa Reddy Bonam [1#], Nicholas C. Hazell [1,2], Mano Joseph Mathew [3,4], Yuejin Liang [1], Xuxiang Zhang [5], Zhi Wei [5], Mohamad-Gabriel Alameh [6], Drew Weissman [6], Haitao Hu [1,7,8,#,!]

1 Department of Microbiology and Immunology, University of Texas Medical Branch, Galveston, TX 77555, USA; bsrpharmacy90@gmail.com (S.R.B.); yu2liang@UTMB.EDU (Y.L.); haihu@UTMB.EDU (H.H.)

2 Department of Pathology, University of Texas Medical Branch, Galveston, TX 77555, USA; nchazell@utmb.edu (N.C.H.)

3 EFREI Research Lab | Panthéon Assas University, 30-32 Avenue de la République, 94800 Villejuif, France

4 Laboratoire Génomique, Bioinformatique, et Chimie Moléculaire, EA7528, Conservatoire National des Arts et Métiers, HESAM Université, 2 rue Conté 75003 – Paris, France; mano.mathew@efrei.fr (M.J.M.)

5 Department of Computer Science, New Jersey Institute of Technology, Newark, NJ 07102, USA; xz445@njit.edu (X.Z.); zhiwei04@gmail.com (Z.W.)

6 Department of Medicine, University of Pennsylvania Perelman School of Medicine, Philadelphia, PA 19104, USA; Mg.Alameh@pennmedicine.upenn.edu (M.G.A.); dreww@pennmedicine.upenn.edu (D.W.)

7 Institute for Human Infections and Immunity, University of Texas Medical Branch, Galveston, TX 77555, USA.

8 Sealy Institute for Vaccine Sciences, University of Texas Medical Branch, Galveston, TX 77555, USA.

# Correspondence: Haitao Hu, PhD; haihu@UTMB.edu; Srinivasa Reddy Bonam, PhD; bsrpharmacy90@gmail.com

! Lead Contact.

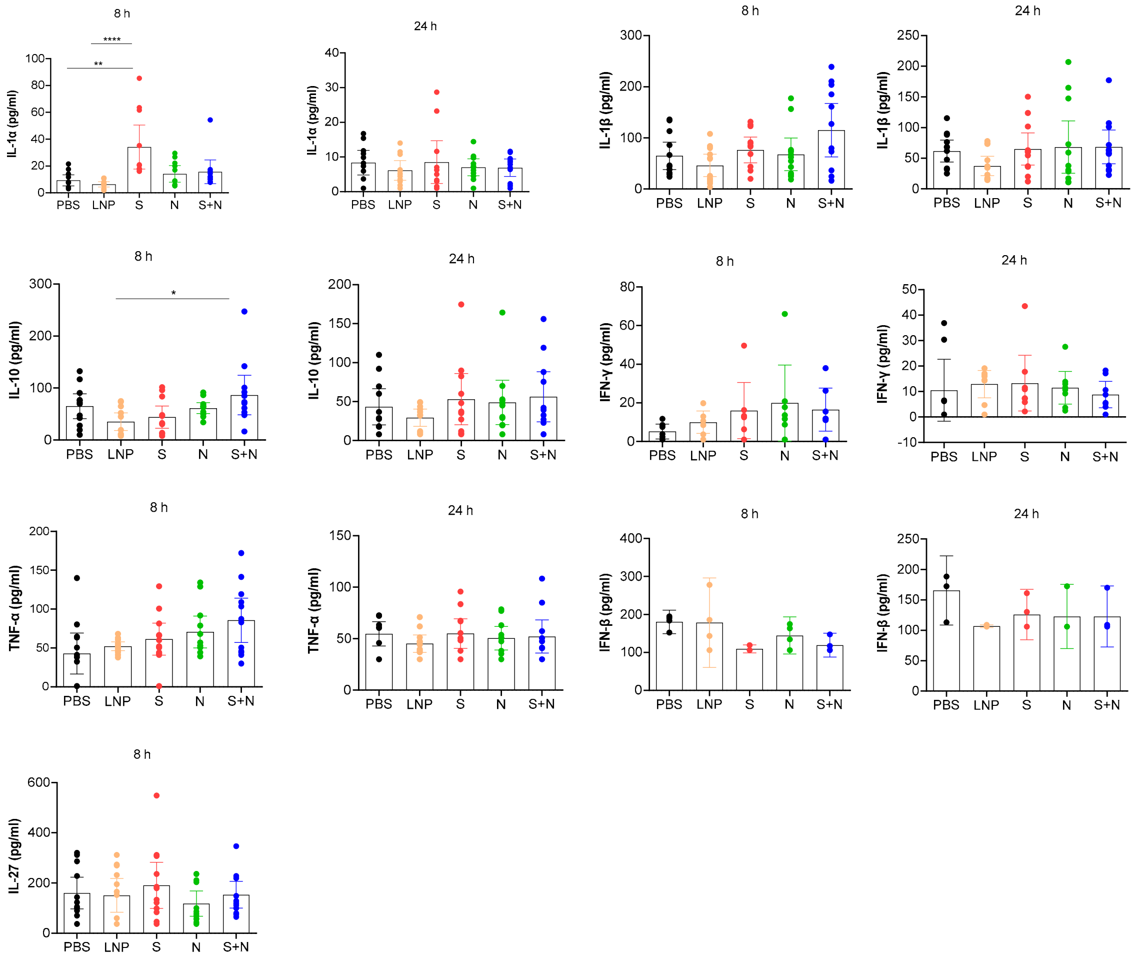

Figure S1. mRNA-LNP vaccination in mice induces robust innate immune response. Balb/c mice were injected with either LNP or mRNA-S or mRNA-N or mRNA-S+N intramuscularly. B. Serum cytokines were measured after 8 and 24 hours; n = 4-12. Nonparametric Tests-Kruskal-Wallis One-Way ANOVA with Dunn's multiple comparisons post hoc test was used for statistical analysis. *P < 0.05.

A

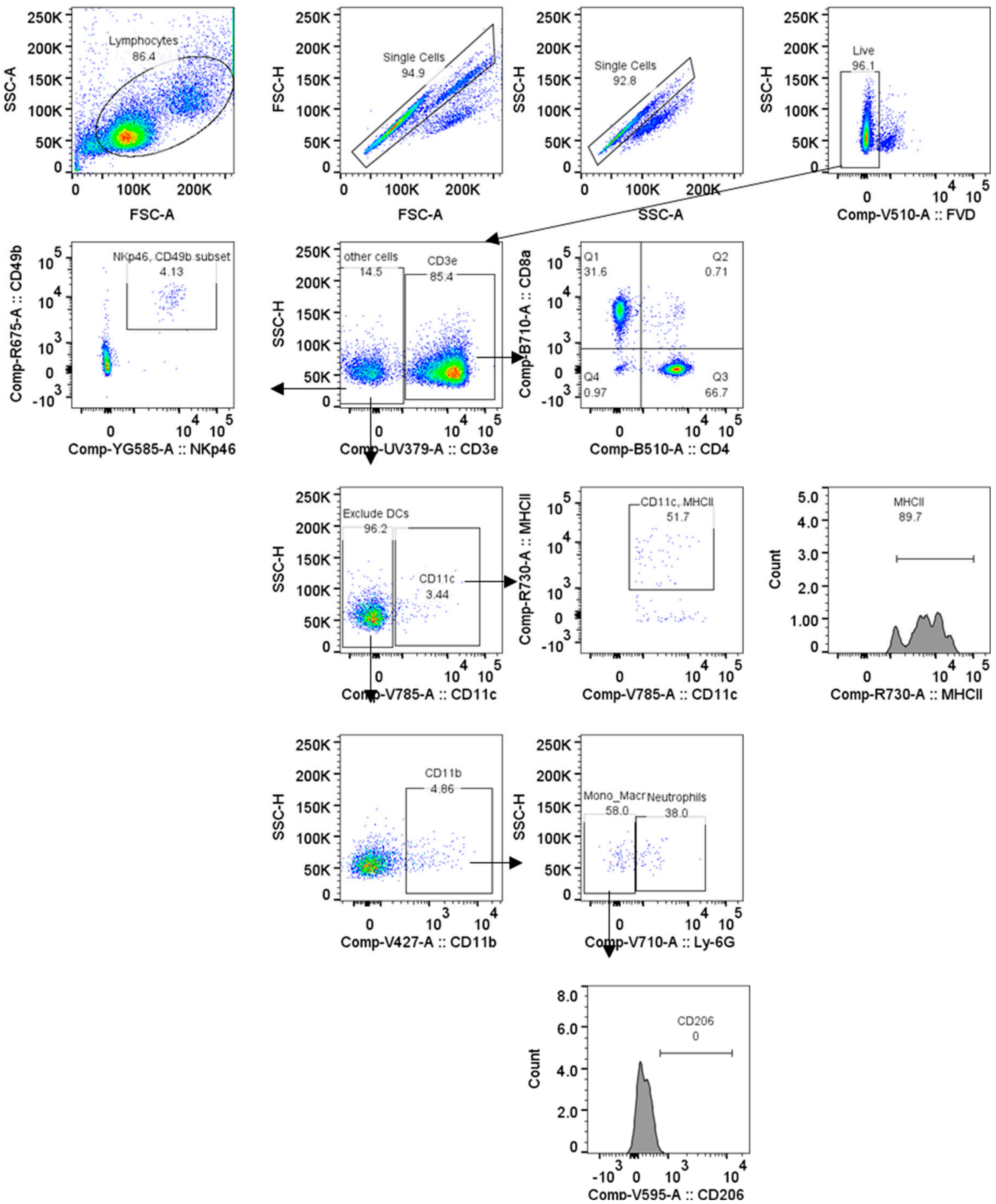

Lymphocytes
86.4
SSC-A
FSC-A
Single Cells
94.9
FSC-H
FSC-A
Single Cells
92.8
SSC-H
SSC-A
Live
96.1
SSC-H
Comp-V510-A :: FVD
NKp46, CD49b subset
4.13
Comp-R675-A :: CD49b
Comp-YG585-A :: NKp46
other cells
14.5
CD3e
85.4
SSC-H
Comp-UV379-A :: CD3e
Q1
31.6
Q2
0.71
Q4
0.97
Q3
66.7
Comp-B710-A :: CD8a
Comp-B510-A :: CD4
Exclude DCs
96.2
CD11c
3.44
SSC-H
Comp-V785-A :: CD11c
CD11c, MHCII
51.7
Comp-R730-A :: MHCII
Comp-V785-A :: CD11c
MHCII
89.7
Count
Comp-R730-A :: MHCII
CD11b
4.86
SSC-H
Comp-V427-A :: CD11b
Mono_Macr
58.0
Neutrophils
38.0
SSC-H
Comp-V710-A :: Ly-6G
CD206
0
Count
Comp-V595-A :: CD206

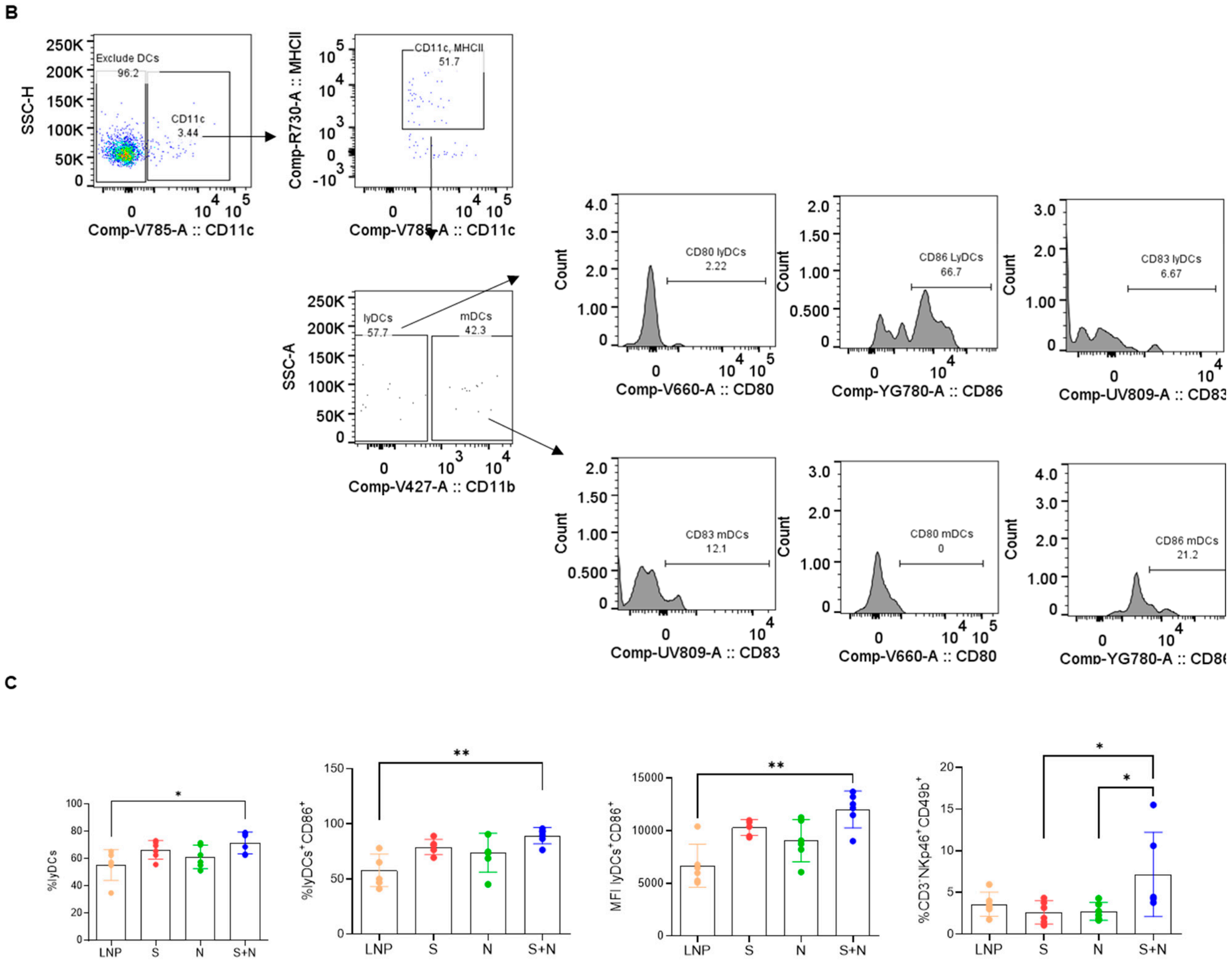

Figure S2. Immune phenotyping of lymph nodes from mRNA-LNP vaccinated mice. A-B. Gating strategies of innate and adaptive immune cells. C. Activation status of DCs and NK cells were measured by co-stimulatory marker CD86 and NKp46⁺CD49b⁺, respectively; n = 4-6. Data were presented as mean with 95% confidence interval. Nonparametric Tests-Kruskal-Wallis One-Way ANOVA with Dunn's multiple comparisons post hoc test was used for statistical analysis. *P < 0.05 and **P < 0.01.

A

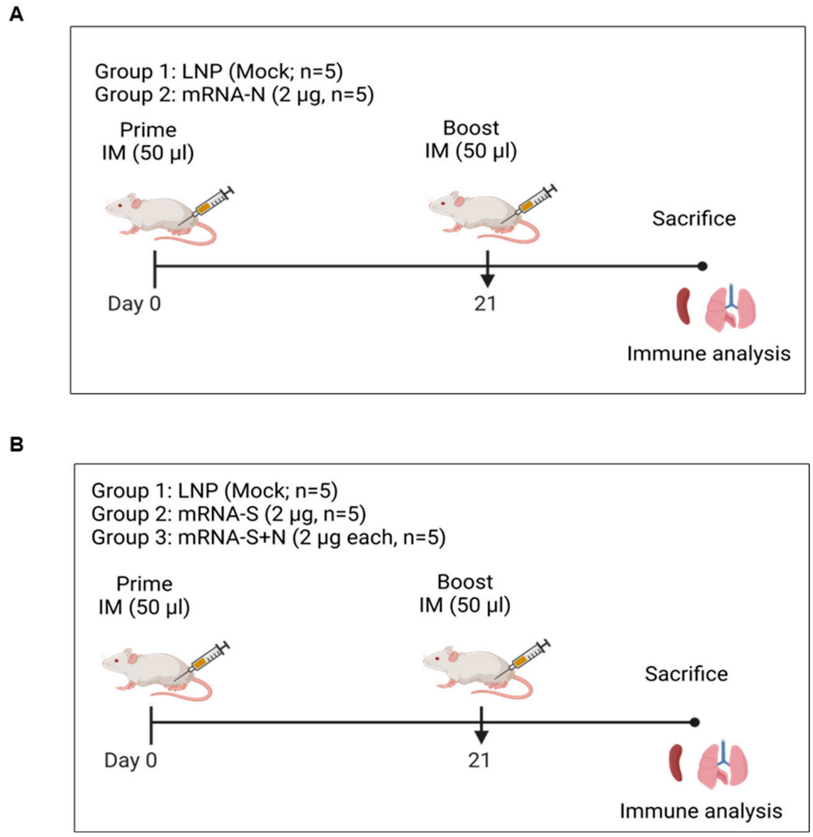

Figure S3. mRNA-LNP vaccination design. A-B. Balb/c mice were injected with either LNP or mRNA-S or mRNA-N or mRNA-S+N intramuscularly (as indicated in the illustration) on day 0, and booster dose at day 21. Two weeks later booster dose, mice were sacrificed and harvested the spleen and lungs.

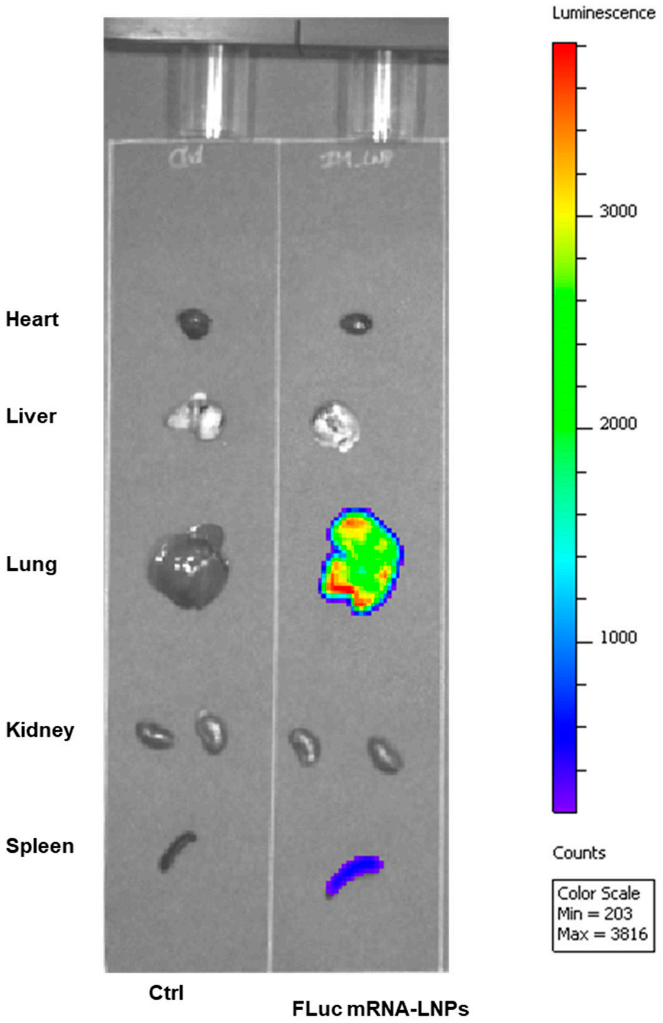

Figure S4. *In vivo* FLuc mRNA-LNP delivery. Representative ex vivo images of heat, lung, liver, spleen, and kidneys collected from either nontreated or FLuc mRNA-LNP treated mice.
